# Notes and News

**Published:** 1894-09-22

**Authors:** 


### Notes and News.

Collected and Communicated.

Sir W. Arrol recently laid the foundation-stone of the Quarrier Consumption Hospital at Bridge of Weir, near Glasgow. The hospital will be the first of the kind in Scotland. It is to be built on the separate house system, to contain 35 to 50 beds. It is hoped the buildings will be ready in a year.

For some time past the very necessary extension of the Bradford Infirmary has been contemplated. The delay has been caused by lack of funds. The committee have now secured £2,000 towards the £55,000 which is required, and as the need of accommodation is pressing, it is proposed to commence the building hoping that the remaining sum requisite will be soon forthcoming. The enlargement will consist of observation wards, out patients' consulting room, mortuary, pathological rooms, and better kitchen premises.

The disease stated to be an epidemic of exfoliative dermatitis, which has shown itself in the infirmaries of several metropolitan workhouses, is now practically over. The cause of the outbreak has not yet been traced, but the medical officers are still investigating the matter. At St. George's-in-the-East condensed milk is now used instead of the usual supply, and although the disease has been in no wise traceable to the milk in use, since the consumption of it has ceased, a marked diminution of cases has been observed.

The new Clinical Research Laboratory is likely to prove of great use to the medical profession. The scheme has the support of many eminent leaders of the profession, amongst whom Mr. Jonathan Hutchinson and Sir W. Broadbent are numbered. The laboratory will be under the direction of Dr. Galloway, Mr. J. Target, M.S., and Mr. F. Hopkins, B.Sc. The scale of charges is very moderate, and will thus afford a saving of time and labour to many busy practitioners. Subscribers have special privileges. Inquiries should be addressed to Mr. Wells, Clinical Research Laboratory, 5, Denman Street, S.E.

Prizes will be distributed to the students of St. Thomas's Medical School on Monday, October 1st, by the Rector of Lincoln College, Oxford.

The Medical School of St. Mary's Hospital will open for the session on Monday, October 1st. The introductory address will be delivered by Dr. Scanes Spicer, in the school, at 4 p.m. on that day.

The International Congress of Hygiene at Buda-Pest is pronounced to have been a success. The two main subjects of general interest were cholera and diphtheria, the antitoxin treatment of the latter being elaborately demonstrated and discussed. The statistics were most striking and encouraging.

Bacteriology, through the agency of Mr. Hankin, a well-known bacteriologist in India, has been most serviceable in tracing the origin of the prevalence of typhoid fever which occurred in a regiment stationed in India. He discovered that dahi, a particular form of curdled milk, was the originator of the mischief. The dahi became contaminated by foul water, and formed a rapid breeding ground for the typhoid germs. Bacteriology has been somewhat impatiently regarded as purely of scientific interest rather than applicable to practical use. Its use in the checking and discovery of impurities, however, is daily demonstrated, and though many germs are yet unconquerable when they have entered the human form, they are to be avoided and kept under to a large extent by the aid of the vigilant bacteriologist.

A systematic inspection of the bakehouses of Poplar is about to be made by the Board of Works. There are several points at issue in the matter. With some people interest will concentrate on the conditions under which those employed in the bakehouses work, whilst to others the all-important question will be that concerned with the purity of the bread itself. The master bakers seem to look upon this latter point as the one which will affect them most, and in a journal devoted to bakers' interests a writer emphasises the fact that great heat sterilises bread. This fact is not borne out in the action of the Municipal Laboratory of Paris, which has prohibited the use of well water in making bread, on account of the contamination which might occur thereby, which heat would not destroy.

About ninety members of the Buda-Pesth Congress arrived in Constantinople on Thursday morning (the 13th), having done 24 hours quarantine in the train. They saw part of the city in the morning, and in the afternoon visited Scutari and the Convent of the Howling Dervishes. On Friday they visited the beautiful Tower of Galata, had an excursion up the Golden Horn, and in the evening they dined in the Municipal Gardens as guests of the Sultan. On Saturday they went to the Museum of Antiquities and the mosques. On Sunday morning the Princes' Islands, in the Sea of Marmora, and in the afternoon an excursion up the Bosphorus, to the entrance to the Black Sea, occupied their time. On Monday morning early they saw the Imperial Treasury, and left by special train later. Some took the Austrian Lloyd steamer to Trieste.

